# The Viral Fraction Metatranscriptomes of Lake Baikal

**DOI:** 10.3390/microorganisms10101937

**Published:** 2022-09-29

**Authors:** Sergey Potapov, Andrey Krasnopeev, Irina Tikhonova, Galina Podlesnaya, Anna Gorshkova, Olga Belykh

**Affiliations:** Limnological Institute SB RAS, 3, Ulan-Batorskaya, 664033 Irkutsk, Russia

**Keywords:** metatranscriptome, viral fraction, Lake Baikal, RNA and DNA viruses

## Abstract

This article characterises viral fraction metatranscriptomes (smaller than 0.2 µm) from the pelagic zone of oligotrophic Lake Baikal (Russia). The study revealed the dominance of transcripts of DNA viruses: bacteriophages and algal viruses. We identified transcripts similar to *Pithovirus sibericum*, a nucleocytoplasmic large DNA virus (NCLDV) isolated from the permafrost region of Eastern Siberia. Among the families detected were RNA viruses assigned to Retroviridae, Metaviridae, Potyviridae, Astroviridae, and Closteroviridae. Using the PHROG, SEED subsystems databases, and the VOGDB, we indicated that the bulk of transcripts belong to the functional replication of viruses. In a comparative unweighted pair group method with arithmetic mean (UPGMA) analysis, the transcripts from Lake Baikal formed a separate cluster included in the clade with transcripts from other freshwater lakes, as well as marine and oceanic waters, while there was no separation based on the trophic state of the water bodies, the size of the plankton fraction, or salinity.

## 1. Introduction

Viral communities in aquatic ecosystems are extremely diverse and include viruses that infect bacteria, archaea, and eukaryotic micro- and macroorganisms. Moreover, there are also virophages, viruses that cannot replicate without another virus, and viroids, subviral agents. Both DNA and RNA genomes can represent viruses. It is still unclear which of them predominates, although there is evidence (measuring RNA concentrations by fluorometry) that RNA viruses can dominate virioplankton [1,2].

Viruses play a key role in aquatic ecosystems by affecting microbial diversity, host metabolism, and nutrient cycle through host lysis [3]. Currently, most research focuses on DNA viruses, including DNA viromes, from aquatic ecosystems. High-throughput sequencing is increasingly being used to study the diversity and functional role of viruses [4,5,6,7].

The first study of freshwater RNA viromes was carried out in the highly eutrophic artificial Lake Needwood (Maryland, MD, USA) in winter and summer [8]. The results indicated that, as with in marine RNA virioplankton, most of the sequences obtained had no clear similarity to known sequences from databases. The samples contained approximately 30 virus families, including plant, insect, vertebrate, and human viruses. The most common were sequences of the order Picornavirales containing positive-sense, single-stranded genomic RNA and infecting a wide range of hosts (vertebrates, invertebrates, protozoa, and plants). However, like marine RNA viromes, none of the sequences was identified to be related to RNA-phages.

A study of Quantuck Bay (dominated by *Aureococcus anophagefferens*) and Narragansett Bay (dominated by diatoms) investigated polyadenylation-selected RNA sequences from microbial communities and indicated severe infection with various giant viruses (NCLDVs). In both bays, the bulk of the assembled contigs belonged to the families Mimiviridae and Phycodnaviridae. A decrease in bloom was accompanied by an increase in the activity of other viruses, including (+) ssRNA viruses. Picornavirales contigs accounted for 62% of the total non-NCLDV viral contigs for Quantuck Bay and 74% of this group for Narragansett Bay [9].

Transcriptomics methods identified both DNA and RNA viruses in viromes from the Baltic Sea and freshwater Lake Tornetrask (Sweden) [10]. Transcripts of viruses belonging to families such as Nanoviridae, Circoviridae, Parvoviridae, and Microviridae were identified among the ssDNA viruses. Double-stranded DNA viruses were found to be more abundant in the small cellular fraction (less than 0.8 to 0.1 µm) and most likely derived from bacteriophages or picoeukaryotic phytoplankton viruses. Using RNA-dependent RNA polymerase (*RdRp*) as a reference marker gene for phylogeny revealed that dsRNA viruses and algal viruses had the greatest taxonomic abundance. There were also Retroviridae (+ssRNA), Picornavirales (+ssRNA), Mononegavirales (−ssRNA), and *Ourmiavirus* (+ssRNA).

A seasonal analysis of the 0.2 to 5 µm fraction in three freshwater lakes (New York, NY, USA) [11] using transcriptomics indicated that among the 30 libraries obtained, only 30 assembled contigs from more than 190 thousand had similarities to RNA viruses. The authors estimated the abundance of four picornavirus genotypes and one reovirus every month from August 2014 to May 2015. Some genotypes of RNA viruses had seasonal trends, but in general they were all present in the three lakes throughout the study period.

The RNA-seq method identified over 4500 new RNA viruses during the study of virioplankton of the Yangshan deep-water harbour near the mouth of the Yangtze River in the seawater–freshwater mixing zone. Based on comparisons of the assembled *RdRp* genes, the majority of the previously unknown viruses corresponded to the kingdom Orthornavirae, with the largest group belonging to the order Picornavirales [12].

The first studies of viruses in Lake Baikal began in the 1990s with a focus on RNA viruses. Genetic and microscopic analyses elucidated the cause of the mass mortality of the endemic Baikal seals (*Phoca siberica*) between 1987 and 1989. A morbillivirus (family Paramyxoviridae) similar to canine distemper virus (CDV) triggered the disease and death of the seals [13,14,15].

Since 2010, viruses in Lake Baikal have continued to be studied mainly using molecular techniques (targeted sequencing and shotgun), first by analysing signature genes of DNA viral communities [16,17,18] and later by high-throughput sequencing for metagenomic analysis of DNA viromes and genome assembly of isolated DNA strains [19,20,21,22,23,24].

Invertebrate transcriptome sequencing confirmed that transcriptomes are an effective tool for extracting viral genomes [25]. Transcriptomes provide an answer to the question of what is happening in the community at the molecular level. In this article, we analyse transcriptomes resulting from the total RNA extracted from a viral (less than 0.2 µm) planktonic fraction of the pelagic zone of Lake Baikal. This study aims to identify the most active viruses, determine the functional affiliation of the dominant transcripts, and obtain the first information about the composition of RNA viruses in Lake Baikal.

## 2. Materials and Methods

### 2.1. Sample Preparation and Sequencing

**Figure 1:**
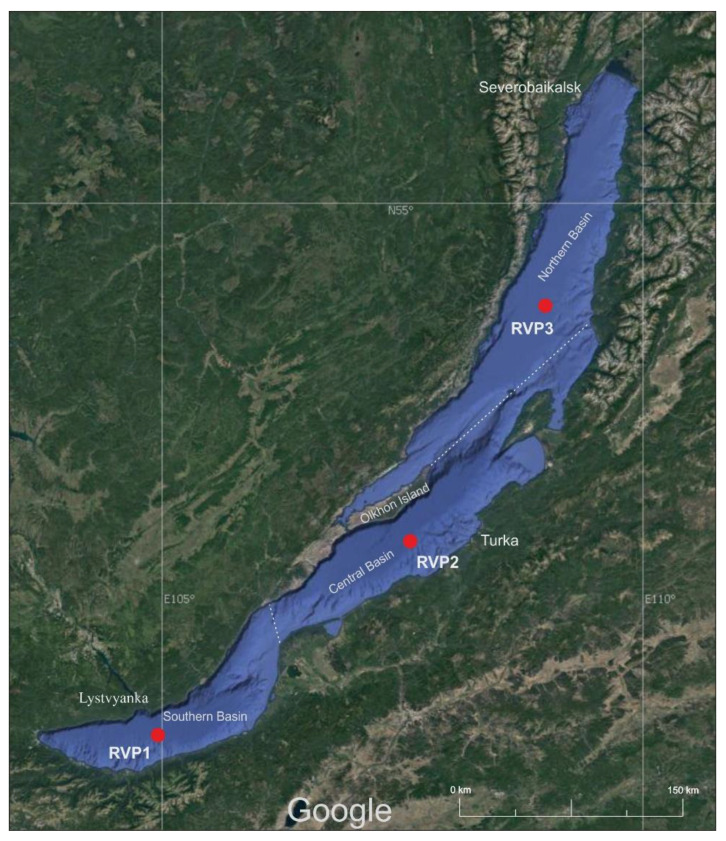
Map of the sampling area. Imagery from 2021 NASA, TerraMetrics, Map data © 2022 INEGI.

Water with a volume of 30 L was sampled in July 2021 at three sites of the pelagic zone of Lake Baikal from the surface to a depth of 50 m (integrated sampling of 5 L from depths of 0, 5, 10, 15, 25, and 50 m). The locations of the sampling sites in three basins of Lake Baikal were as follows: RVP1—central station “Listvyanka settlement–Tankhoy settlement” (51°41.187 N, 105°00.096 E), RVP2—central station “Ukhan Cape–Tonky Cape” (52°53.117 N, 107°30.745 E), and RVP3—central station “Elokhin Cape–Davsha settlement” (54°25.531 N, 109°01.431 E) (Figure 1); RVP—RNA Virus Point (from 1 to 3).

The samples were filtered stepwise through polycarbonate filters with a pore size of 0.4 µm and 0.2 µm (Sartorius, Göttingen, Germany) to remove debris and zoo-, phyto-, and bacterioplankton. The filtrate of each sample was concentrated to 100 mL using a Vivaflow 200 tangential filtration system (Sartorius, Göttingen, Germany), then to 1 mL using Vivaspin Turbo 15 microcentrifuge tubes (Sartorius, Göttingen, Germany) (50 kDa) at 4 °C and 3000 rpm. The concentrate was frozen in liquid nitrogen and stored at −70 °C until further analysis.

Total RNA was extracted with ExtractRNA (Evrogen, Moscow, Russia) according to the protocol. For the RNA-seq library preparation according to the MGIEasy RNAseq Library Prep Set protocol (MGI Tech, Shenzhen, China), 100–200 ng of extracted RNA was used. The following stages were performed: RNA fragmentation, reverse transcription, synthesis of the second strand, end-polishing of dsDNA fragments, and adapter ligation (containing 10 bp single-end indexes). A DNBSEQ-400 platform (MGI Tech, Shenzhen, China) with pair-end reads (2 × 150) was used for sequencing. Sequencing was carried out at the “Core Sequencing Centre” of the Kurchatov Centre for Genome Research, Moscow, Russia.

### 2.2. Bioinformatic Analysis

Fastq files were checked using FastQC v. 0.11.9 [26]. The reads were assembled by the metaSPAdes v. 3.15.0 (Center for Algorithmic Biotechnology, Saint Petersburg, Russia) assembler [27]. Contig coverage was performed in BWA-mem v. 0.7.17; statistical analysis was carried out in Samtools v. 1.9 [28]; blastn v. 2.12.0 + (e-value 10^−^^5^) was used for taxonomic annotation of contigs (only more than 500 bp), RefSeq database (release 211); the results were visualised in BlobTools v. 1.1.1 (Institute of Evolutionary Biology, Edinburgh, UK) [29]. Open reading frames (ORF) were determined in GeneMarkS v. 3.36 (Georgia Institute of Technology, Atlanta, GA, USA) [30]. ORF taxonomic annotation was performed in Diamond v. 2.0.15 [31] with the *-more-sensitive* and *-min-score 50* parameters using the RefSeq (release 211) and NR (release 243) databases; sequences with protein identity levels ≥35% were selected for the analysis.

Functional analysis was performed using the PHROG database v. 3 [32], the Virus Orthologous Groups Database (VOGDB) version 213 (https://vogdb.org/) (accessed on 10 January 2022), and the SEED subsystems (https://pubseed.theseed.org/) (accessed on 10 January 2022).

Functional annotation of the genes was performed using Diamond with the *-more-sensitive*, *-min-score 50* parameters and the PHROG database, followed by normalisation (“total” method) and visualisation in the R programming language using the vegan v. 2.5-7, gplots v. 3.1.3, and viridis v. 0.6.2 packages.

Annotation of proteins according to the database SEED was performed using the program Super-Focus [33] (aligner—rapsearch, cluster size database—100), program version 0.34. Since this database does not contain only viral proteins, our contigs (more than 500 bp) were sorted using the program Virsorter2 [34], and calculations were performed at https://cyverse.org/ (accessed on 5 May 2022) [35]. Open reading frames (ORF) were determined in GeneMarkS.

Protein identification using the VOG database was performed with the HMMER 3.2.1 program (http://hmmer.org/) (accessed on 3 June 2022) using hmmsearch (threshold e-value 10^−5^).

The auxiliary metabolic genes (AMG) were searched using the Vibrant v. 1.2.1 tool (University of Wisconsin–Madison, Madison, WI, USA) [36]. The proteomic tree was reconstructed using the ViPTree server [37].

For comparative analysis (UPGMA), data from different ecosystems were selected: Lake Seneca, Lake Owasco, Lake Cayuga [11], Lake Tai [38], Tiana Beach, Quantuck Bay [39], Lake Tornetrask, the Baltic Sea [10], the Pacific Ocean [40], and Yangshan deep-water harbour [12]. The raw data were processed in Trimmomatic v. 0.36 [41] and assembled with metaSPAdes v. 3.15.0; ORFs were determined using GeneMarkS v. 3.36; annotation was carried out in Diamond (*-more-sensitive*, *-min-score 50*, RefSeq database); sequences with a protein identity level ≥35% were selected for the analysis. The distance matrix was based on the abundance of viral proteins using the vegan v. 2.5-7 and gplots v. 3.1.3 packages implemented in the R software (v. 4.1.3); data was normalised by “total”, dissimilarity matrix—method “bray”.

All calculations were performed on HPC-cluster “Akademik V.M. Matrosov” (“Irkutsk Supercomputer Centre of SB RAS, http://hpc.icc.ru” (accessed on 4 August 2022)).

## 3. Results

### 3.1. Taxonomic Annotation of Transcripts

**Figure 2:**
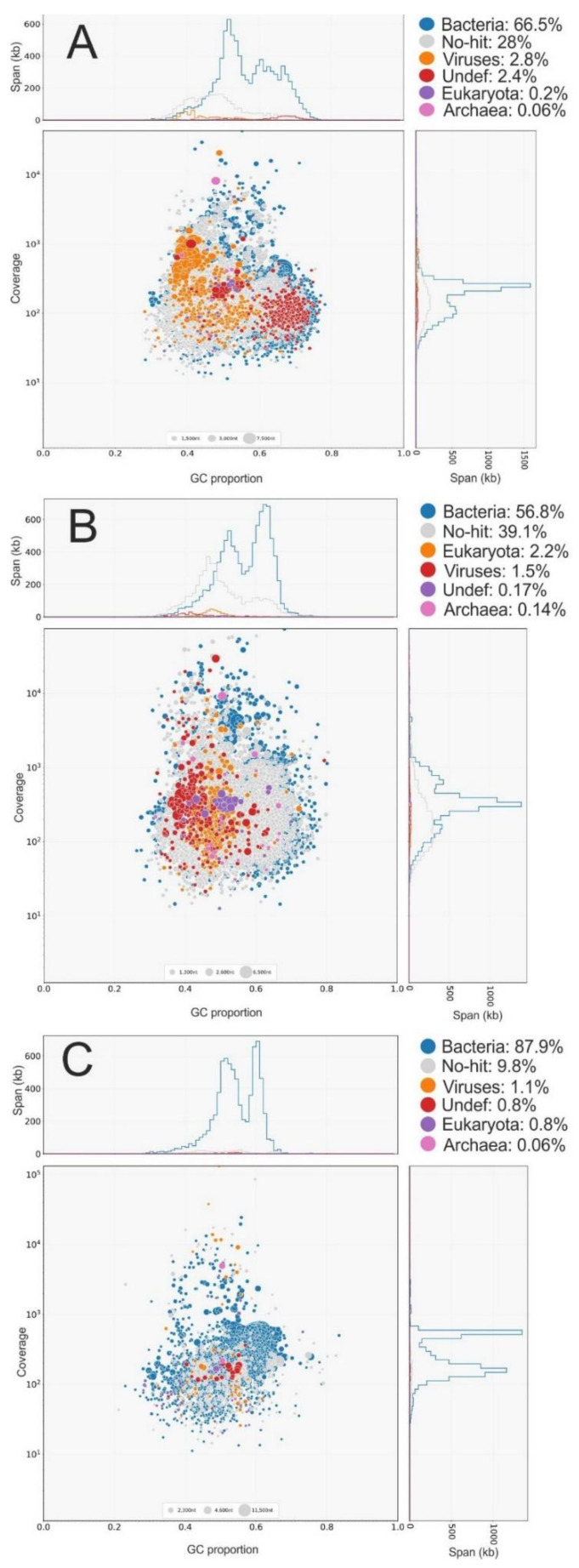
Coverage, GC content, and taxonomy (RefSeq) of contigs from metatranscriptomes. (**A**)—RVP1, (**B**)—RVP2, and (**C**)—RVP3.

As a result of sequencing, we obtained the following number of reads: RVP1—24.6 million reads, RVP2—47.5 million reads, and RVP3—21.5 million reads. Based on the blastn analysis, the bulk of the identified sequences belonged to bacteria (from 56.8% to 87.9%) (Figure 2); the proportion of viral sequences ranged from 1.1% to 2.8%. The low number of viral sequences is usual for environmental samples and is related to the difficulty of differentiating and extracting sequences belonging only to viruses [10,42]. In addition, some sequences (9.8 to 39.1%) had no similarity to the sequences known from the RefSeq database; this was the so-called “dark matter”. Apparently, bacteria of small sizes passed through the filter with a pore size of 0.2 µm despite pre-filtering. This phenomenon was due to the presence of ultramicrobacteria in Lake Baikal and probably also to the presence of dissolved nucleic acids in the water [43].

The GC content in contigs belonging to viruses was 44–49%; in bacterial sequences it was 52–58%; in eukaryotic sequences it was 46–49%; and in archaeal sequences it was 49–53%.

**Figure 3:**
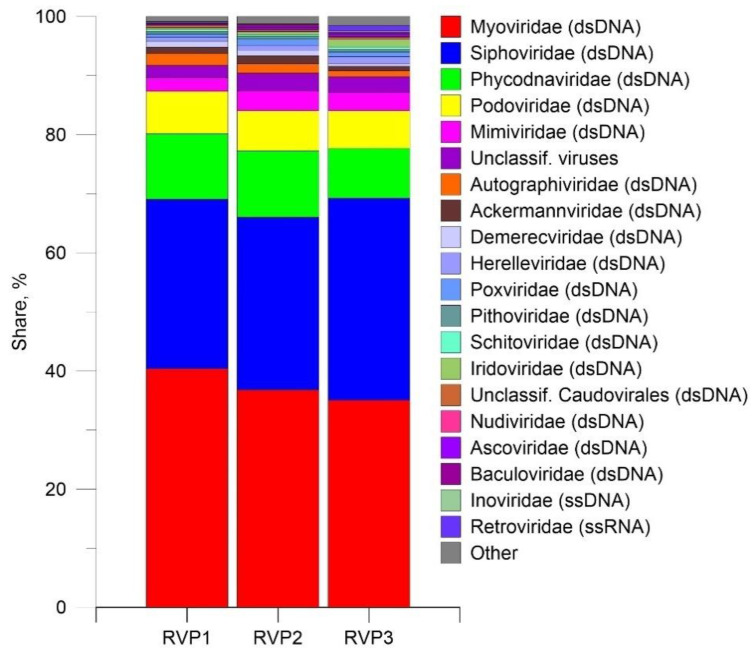
Abundance of viral taxa at the family level in the transcriptomes of Lake Baikal.

The identification of taxonomic ORFs using the RefSeq NCBI protein base indicated that the most representative viral ORFs belonged to the DNA families Myoviridae (35–40.4%) and Siphoviridae (28.7–34.2%) (Figure 3). In general, the bulk of viral transcripts was attributed to bacteriophages; the family Phycodnaviridae represented 8.4–11.2%; the contribution of the family Mimiviridae reached 2.3–3.4%. In transcriptomes, there were ORFs assigned to RNA viruses of the families Retroviridae (0.09–0.9%), Metaviridae (0.06–0.4%), Potyviridae (0.02–0.04%), Astroviridae (0.02%), and Closteroviridae (0.01%).

Among the retroviral-like ORFs (67 in total), we detected the following:

- putative viral DNA polymerase (YP_009243641), identity 50–71%, lowest e-value 2.7 × 10^−^^32^, ORF length 147–327 nt, transcript belongs to *Bovine retrovirus* CH15 (*Betaretrovirus*) hosted by large cattle;
- pol protein (YP_009513211), identity 46.3–64.7%, lowest e-value 5.0 × 10^−^^22^, ORF length 198–231 nt, similar to Koala retrovirus (*Gammaretrovirus*) according to the annotation;
- ubiquitin-like protein (NP_598374), identity 58.6%, e-value 2.3 × 10^−^^12^, closest relative is *Murine osteosarcoma virus* (*Gammaretrovirus*), natural hosts are mice;
- gag protein (NP_056901), identity 100%, e-value 3.6 × 10^−^^62^, ORF length 231–288 nt, the closest relative is equine infectious anaemia virus (Orthoretrovirinae), infects members of the horse family (Equidae) and others.

Two proteins represented ORFs similar to the family Metaviridae (42 in total): reverse transcriptase (YP_009666308, *Cladosporium fulvum* T-1 virus) and ORF B (YP_009507248, *Trichoplusia ni* TED virus). The identity varied from 35.3% to 56.4% (reverse transcriptase) and from 38.2% to 52.5% (ORF B); the lowest e-values were 8.9 × 10^−^^51^ and 2.8 × 10^−^^22^, respectively. The length of the nucleotide sequences varied from 153 to 537 nt for reverse transcriptase, and from 144 to 543 nt for ORF B. *Cladosporium fulvum T-1 virus* (ssRNA) infects the *Cladosporium fulvum* fungus that parasitises tomato and other nightshade crops. *Trichoplusia ni* TED virus has an unsegmented single-stranded RNA genome and is hosted by *Trichoplusia ni*, a medium-sized moth of the family Noctuidae.

The HAM1-like protein represents the family Potyviridae, the closest relative of which were Cassava brown streak virus (YP_007032446) and Ugandan cassava brown streak virus (YP_004063983). The family Potyviridae contains positive-sense RNA viruses and includes more than 30% of known plant viruses. Among the closest relatives of the family Astroviridae, there was a capsid protein (YP_009275018), and of the family Closteroviridae, an unknown protein with a molecular weight of 59 kDa (NP_813799).

The identified sequences of viruses that infect fish and humans were insignificant. For example, Poxviridae-like (0.5–0.8%) sequences (viruses of this family infect animals) were represented by *Anomala cuprea entomopoxvirus* (infect shining leaf chafers, *Anomala*), *Diachasmimorpha entomopoxvirus* (hosted by parasitoid wasps of the family Braconidae), and Salmon gillpox virus (infect gill epithelial cells of the Atlantic salmon). Among the neighbours detected, there was the family Iridoviridae (0.3–1.3%), represented by Lymphocystis disease virus, which causes a widespread viral disease of freshwater and marine fish. *Anguillid herpesvirus 1* represented the family Herpesviridae (0.09–0.1%). The family Retroviridae (0.09–0.9%) included different species, including Atlantic salmon swim bladder sarcoma virus, Baboon endogenous virus, and human endogenous retrovirus K. Other members of the families that infect humans, birds, pigs, and fish (Alloherpesviridae, Circoviridae, Asfarviridae, Astroviridae, and Papillomaviridae) accounted for less than 0.1%. This study revealed insignificant amounts of the short sequences of Papillomaviridae, namely, human papillomavirus 9 (231 nt, protein similarity—85.7%), Herpesviridae: human gammaherpesvirus 4 (243 nt, protein similarity—59.8%), and human betaherpesvirus 5 (207 nt, protein similarity—48.5%), and Poxviridae, namely, Akhmeta virus (171–612 nt, protein similarity—42.3–48.7%), Yaba-like disease virus (171–189 nt, protein similarity—40.4–50%), and Yaba monkey tumour virus (252 nt, protein similarity—40.7%).

Interestingly, the family Pithoviridae (DNA viruses) was present in the Baikal samples, the sequences of which were similar to the sequences of recently detected *Pithovirus sibericum* isolated from 30,000-year-old permafrost [44]. The RVP1 transcriptome had 16 proteins similar to the *Pithovirus sibericum* proteins (35.2–56.4% identity), while RVP2 had 20 (36.1–50.9% identity), and RVP3 had 8 (35.6–60% identity). Additionally, we identified proteins belonging to *Cedratvirus A11* from the same family: RVP1—34 (35.1–56.9% identity), RVP2—26 (35.1–57.7% identity), and RVP3—13 (37.3–57.4% identity) (Table 1).

**Figure 4:**
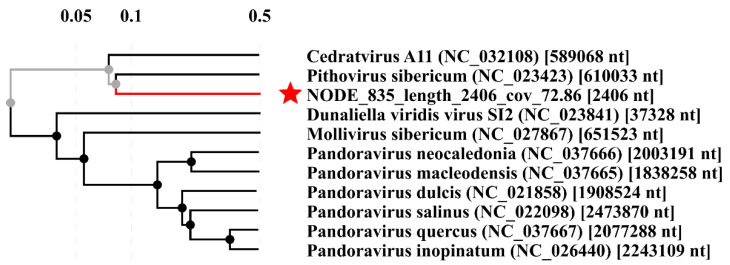
Proteomic tree reconstructed with the Node_835 (RVP3, the sequence is marked with an asterisk) using the ViPTree server.

The proteomic tree with the sequence of RVP3 Node_835 (2406 nt), which is similar to adenylosuccinate synthetase (YP_009000992), showed inclusion in the cluster, which may again indicate the affiliation of this sequence with the virus (Figure 4).

In the samples, according to blastX analysis (RefSeq database), most sequences belonged to the DevA family ABC transporter ATP-binding protein (Planktothrix phage PaV-LD, accession number YP_004957306), with similarity at the amino acid level ranging from 35.2 to 46%. The cyanophage PaV-LD was isolated from the shallow freshwater Lake Donghu (China); its host is the filamentous cyanobacterium *Planktothrix agardhii* [45]. No representatives of this genus are known in Lake Baikal, but there are closely related genera from the Microcoleaceae family [46].

Sequences similar to Yellowstone Lake phycodnavirus were found in RVP samples. Similarity at the amino acid level ranged from 35.4 to 99.5% (RefSeq). Most of them are classified as hypothetical proteins. Among the putative proteins found were the following: (1) ribonucleotide reductase large subunit (YP_009174518)—this enzyme converts ribonucleotides into deoxyribonucleotides, building blocks for DNA replication and repair; (2) DNA polymerase (YP_009174598)—an enzyme involved in DNA replication; (3) GDP-mannose 4,6-dehydratase (YP_009174664, YP_009174611)—the enzyme belongs to the hydrolyases that cleave carbon–oxygen bonds; (4) DNA topoisomerase II (YP_009174603, YP_009174654)—alters the topology of DNA, causing transient double-strand breaks in DNA; (5) major capsid protein (YP_009174377, YP_009174478, YP_009174294, YP_009174363), prolyl 4-hydroxylase (YP_009174672)—catalyzes the formation of 4-hydroxyproline in collagens and other proteins with collagen-like amino acid; (6) FAD-dependent thymidylate synthase (YP_009174300)—plays a central role in the biosynthesis of thymidylate, an important precursor of DNA biosynthesis; and (7) PhoH-like protein (YP_009174586)—cytoplasmic protein and predicted ATPase that is induced by phosphate starvation, heat shock protein 40 (YP_009174434). Yellowstone Lake phycodnaviruses are algal-infecting large dsDNA viruses; four partial genomes were found in the Yellowstone Lake metagenome dataset [47]. The high degree of similarity and coverage suggests the presence of similar giant viruses in Lake Baikal, the investigation of which requires a separate study.

### 3.2. Functional Analysis

#### 3.2.1. PHROG Database

Using the PHROG cluster database of phage proteins, we identified 10 categories: “Unknown function” (43.5–49.8%); “DNA, RNA, and nucleotide metabolism” (14.7–17.7%); “Other” (10.3–10.8%); “Head and packaging” (5.5–9.5%); “Host genes” (AMG, etc.) (7.7–9.3%); “Tail” (2.6–4.5%); “Integration and excision” (2.4–4.3%); “Lysis” (1.0–1.4%); “Transcription regulation” (1.1–1.4%); and “Connector” (0.6–0.9%) (Supplementary Materials, Figure S1). The total number of proteins identified was as follows: RVP1—21854, RVP2—20,082, and RVP3—10587. The category “DNA, RNA, and nucleotide metabolism” included synthesis genes of, e.g., nucleotides or DNA modifications (DNA adenine methylase). The analysis showed that the functional categories had almost the same ratios in the three samples despite the geographical remoteness of the sampling stations. The category with unknown functions was the largest, which was to be expected, as many proteins in databases were not annotated. The category “Host genes (AMG, etc.)” are the genes unnecessary for the phage life cycle, which mainly originate from the host. “Other” are those that did not fit into the other categories.

There were 43.2 to 49.7% of the identified proteins with unknown functions. ABC transporter, terminase large subunit, transposase, and DNA helicase were the most numerous proteins from the closest relatives in the three transcriptomes (Supplementary Materials, Figure S2).

In the RVP1 transcriptome, 829 (3.8%) ORFs resembled the ABC transporter cluster; 578 (2.6%) ORFs corresponded to the terminase large subunit; 498 ORFs (2.3%) belonged to transposase, an enzyme that binds single-stranded DNA and integrates it into genomic DNA, and 469 (2.1%) ORFs corresponded to DNA helicase. In RVP2, 909 (4.5%) ORFs were similar to the ABC transporter cluster, 444 (2.2%) ORFs to the terminase large subunit, 333 (1.7%) ORFs to transposase, and 454 (2.3%) ORFs to DNA helicase. In RVP3, 533 (5%) ORFs were assigned to the ABC transporter cluster, 175 (1.7%) ORFs to the terminase large subunit, 343 (3.2%) ORFs to transposase, and 198 (1.9%) ORFs to DNA helicase.

#### 3.2.2. VOG Database

According to the database VOG, the following numbers of proteins were found in each of the transcriptomes: RVP1-6319, RVP2-5562, and RVP3-2741 (Supplementary Materials, Table S1). Most of the proteins (46–54%) belonged to the “Function unknown” category, which makes it impossible to interpret the results at this stage. Hypothetical proteins occupied between 14.7% and 25%. The most numerous proteins identified were Probable iron transport system ATP-binding protein HI 0361 (VOG19828), BPT4 ATP-dependent DNA helicase uvsW (VOG00561), BPT4 Exonuclease_subunit 2 (VOG00052), and dTDP-glucose 4,6-dehydratase 2 (VOG00143). According to the functional categories of VOGDB, 12 to 14% belonged to “Virus replication”, 7.3 to 12.4% to “Virus structure”, 38 to 48% to “Virus protein with function beneficial for the host”, and 5.7 to 6.6% to “Virus protein with function beneficial for the virus”.

#### 3.2.3. SEED Subsystems

**Figure 5:**
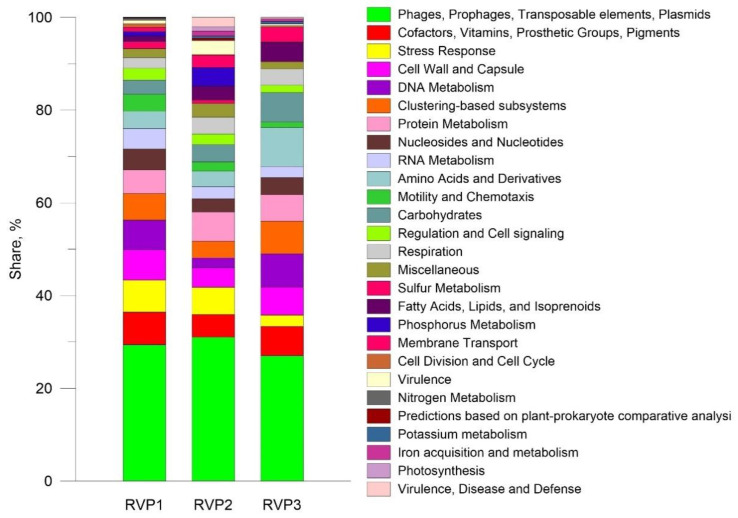
Functional annotations of the RVP1, RVP2, and RVP3 transcriptomes by SEED subsystems.

For the annotation of the viral proteins, the SEED subsystems database was used, and ORFs (amino acids), defined in contigs, which were identified using VirSorter2 (only contigs with more than 500 bp) were taken. The annotation results are shown in Figure 5.

Most viral transcripts were assigned to the category “Phages, Prophages, Transposable elements, Plasmids”, RVP1-29.4%, RVP2-31%, RVP3-27%. This category included discovered proteins such as phage terminase large subunit, integrase, exonuclease, major capsid protein, tail fibre protein, endolysin, etc. The second largest group in RVP1 was “Cofactors, Vitamins, Prosthetic Groups, Pigments” (7%). This category includes the most numerous proteins: probable iron binding protein from the HesB IscA SufA family and Molybdopterin molybdenumtransferase (MoeA). In RVP2, the category “Protein Metabolism” (6.3%) ranked the second, with Translation initiation factor 1 and ATP-dependent Clp protease proteolytic subunit predominating. In RVP3, the second largest category was “Amino Acids and Derivatives” (8.4%), which included proteins such as 2-iminobutanoate/2-iminopropanoate deaminase RidA and Methionine ABC transporter ATP-binding protein.

#### 3.2.4. AMG Genes in Metatranscriptomes

In contigs belonging to bacteriophages, the Vibrant tool detected the following number of AMG genes: RVP1—13, RVP2—4, and RVP3—2 (Table 2).

### 3.3. Comparative Analysis of Transcriptomes

**Figure 6:**
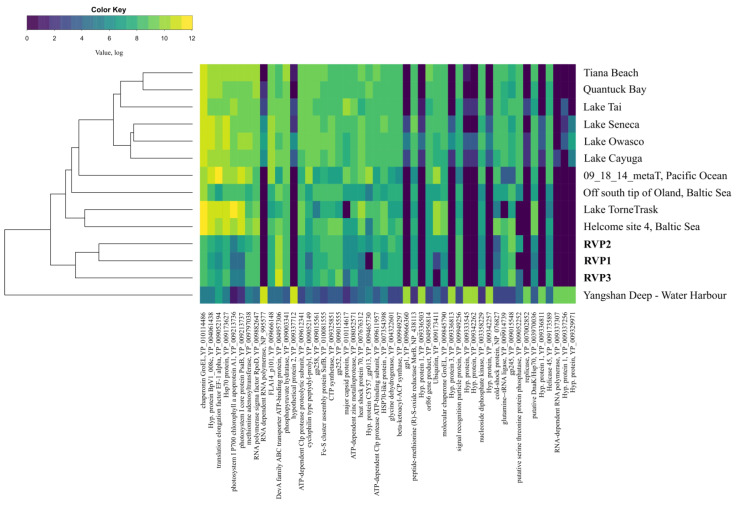
UPGMA dendrogram with a heat map created based on the abundance of viral proteins (RefSeq database); taxa with similarity ≥35% were taken into the analysis.

Samples from Lake Baikal had an isolated cluster that was part of a common cluster with transcriptomes from Lakes Cayuga, Owasco, Seneca, Tornetrask, and Tai, as well as Tiana Beach, Quantuck Bay, the Pacific Ocean (California coast), and the Baltic Sea (Figure 6). Furthermore, RVP1 and RVP2 were more similar to each other. Finger Lakes (Cayuga, Owasco, and Seneca) formed a co-cluster with the samples from Tiana Beach, Quantuck Bay, and Lake Tai. The sample from the Yangshan deep-water harbour was the most distant branch.

A comparative analysis of the transcriptomes did not reveal a clear division into marine and freshwater ones. For example, in our previous study on DNA viromes, samples from Lake Baikal were clustered with freshwater ecosystems [20].

### 3.4. Transcripts of Putative Hosts

**Figure 7:**
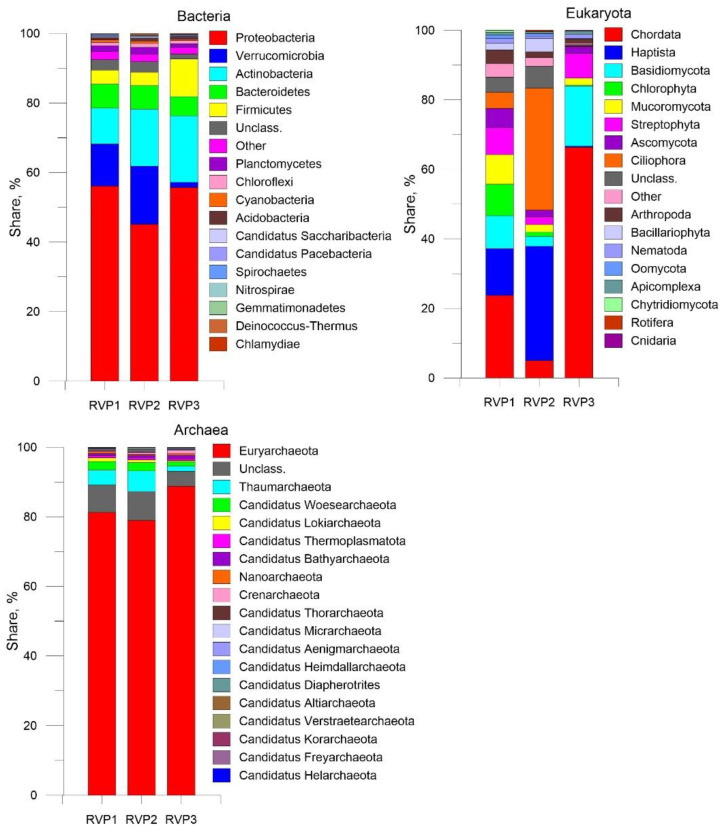
Abundance of taxa from three domains at the phylum level in the three metatranscriptomes.

Based on the NR (NCBI) database, the phyla of the domain Bacteria predominated in the viral fraction transcriptomes: Proteobacteria (45.1–56.1%) and Verrucomicrobia (12.5–16.7%); in RVP3, this phylum was only 1.5%, Actinobacteria was 10.3–19.1%, and Bacteroidetes was 5.5–6.9% (Figure 7). Overall, we identified 127,749 bacterial transcripts in RVP1, 139,583 in RVP2, and 74,266 in RVP3. The bacterial community composition and structure of the viral fraction were similar to those of the bacterial fraction from the pelagic zone of Lake Baikal [48]. Among the closest relatives, the maximum number of unique proteins was 46 in RVP1 (putative AN484_22050, OBQ40850, *Aphanizomenon flos-aquae* WA102), 64 in RVP2 (LEPR-XLL domain-containing protein, WP_104798714, *Limnohabitans* sp. TS-CS-82), and 87 in RVP3 (DUF1725 domain-containing protein, WP_215727872, *Mycobacterium tuberculosis*). The phylum Cyanobacteria was represented by the dominant Eastern Siberia species *Aphanizomenon flos-aquae* (RVP1—0.2%, RVP2—0.3% and RVP3—0.04%) and *Synechococcaceae bacterium* WB6_1A_059 (RVP1—0.04%, RVP2—0.03%, and RVP3—0.01%) in percentage of all bacterial species. It is likely that the low proportion of Verrucomicrobia, which belong to the chemoorganoheterotrophic organisms, in sample RVP3 and the high proportion in RVP1 and RVP2 are due to the fact that the southern and central basins are under the influence of the Selenga River, i.e., associated with a large supply of organic material, especially polysaccharides [49]. RVP3 is the northern basin, the central station “Elokhin Cape-Davsha settlement”, which is furthest away from the influence of the river. Moreover, the seasonal and spatial distribution (in the water area of Lake Baikal) of bacterial phyla is extremely heterogeneous, as are other biota components, e.g., the composition and abundance of phytoplankton, bacterioplankton, autotrophic picoplankton, and ciliates [50,51,52].

The number of transcripts belonging to eukaryotes was as follows: RVP1—2814, RVP2—11,868, and RVP3—8246. The dominant taxa were Chordata (23.7%), Haptista (13.5%), Basidiomycota (9.4%), and Chlorophyta (9.0%) in RVP1; Ciliophora (35.1%), Haptista (32.9%), and Chordata (5.0%) in RVP2; and Chordata (66.3%), Basidiomycota (17.3%), and Streptophyta (7.0%) in RVP3. The predominance of the Ciliophora transcripts in RVP2 is remarkable; the family Oxytrichidae dominated this phylum (72% of all Ciliophora families) with the species *Stylonychia lemnae*.

Based on the transcript annotation results, *Chrysochromulina tobinii* (12.6%) was the most abundant species in RVP1, *Chrysochromulina tobinii* (30.8%) and *Stylonychia lemnae* (25.3%) in RVP2, and *Nyctereutes procyonoides* (13.4%) in RVP3. The similarity to the transcripts of *Nyctereutes procyonoides* (common raccoon dog) was likely associated with the presence of a closely related organism.

Most transcripts belonging to the archaeal domain were similar to the phylum Euryarchaeota (RVP1—81.3%, RVP2—78.9%, and RVP3—88.8%). *Natronomonas* sp. LN261 were the most numerous taxa at the species level (RVP1—12.5%, RVP2—14.3%, and RVP3—21.4%).

## 4. Discussion

Analysis of transcriptomes from Lake Baikal revealed that up to 89% of all transcripts belonged to bacteria, eukaryotes, and archaea. Since we used a viral fraction (smaller than 0.2 µm) in this study, it is very likely that these transcripts are represented by environmental RNA (eRNA) or belong to ultramicrobacteria or viruses that contained host genes (e.g., AMG).

As summarised in the previous overview [53], the fraction and method chosen can affect the results. For example, removal of the cell fraction excludes RNA viruses that lack a capsid, do not pass through the extracellular state, and are vertically transmitted. Single-stranded DNA viruses require a special extraction technique and sequencing approach, such as the use of an additional enzyme (adaptase) during library preparation [54]. Viruses with dsRNA may be underrepresented in RNA viromes due to inefficient conversion to cDNA during sequencing library preparation.

It was difficult to assess the species membership of viruses because (i) some sequences did not have 100% coverage with the reference protein and (ii) could be due to the presence of unknown viruses (with highly divergent sequences), i.e., missing from the databases.

Currently, the taxonomy of viruses is constantly being revised based on new findings. For instance, the class Caudoviricetes includes 33 families, and the long-known families Myoviridae, Siphoviridae, and Podoviridae have been revised and have disappeared [55]. However, the databases are not updated as rapidly as the taxonomy of viruses; therefore, in this study, we use the previous nomenclature (Virus Taxonomy: 2020 Release).

The dominance of bacteriophage transcripts was not a surprise, as previous studies of Lake Baikal using metaviromics had revealed the predominance of phage families over others [19,20,22,56].

Taxa belonging to RNA viruses were insignificant, namely, Retroviridae (0.09–0.9%), Metaviridae (0.06–0.4%), Potyviridae (0.02–0.04%), Astroviridae (0.02%), and Closteroviridae (0.01%), which is related to the difficulty of transcript differentiation and may also be due to the depth of reading. Nevertheless, the data obtained provide an initial understanding (first data) of the taxonomic composition of RNA viruses in Lake Baikal.

The similarity of the transcripts to *Pithovirus sibericum* is interesting. Members of the family Pithoviridae (phylum Nucleocytoviricota) are known as the nucleocytoplasmic large DNA viruses or giant viruses, a group of families of eukaryotic viruses. Currently, the ICTV has not approved this family; in this article, we refer to the NCBI databases. *Pithovirus sibericum* was isolated in 2014 from 30000-year-old permafrost in northeastern Siberia [44]. *Pithovirus sibericum* is a virus with a length of 1.5 µm and a diameter of 500 nm, which has a genome size of 610 thousand base pairs (kb). The first *Cedratvirus A11* (LT671577) (a laboratory strain of *Acanthamoeba castellanii* host) was isolated from environmental samples in Algeria [57], with a virion length reaching 1.2 µm and a maximum diameter of 500 nm; the genome size was 589 kb. The presence of these transcripts in the sample filtered through a 0.2 µm filter could be due to the presence of dissolved nucleic acid in the water.

In terms of functional potential, the bulk of the viral transcripts (with the exception of the unknown ones) was categorised as replication. ABC transporters (transport ATPases) belong to the translocases. These proteins contribute to the movement of molecules mainly through the cell membrane. They catalyse the movement of ions or molecules through membranes or their separation within membranes. Some viruses have genes that encode proteins with membrane transport functions [58]. Recently, 18 different types of putative membrane transport proteins have been identified using the NCBI databases, indicating that they are not rare in viral genomes [59]. These proteins belong mainly to large DNA viruses and bacteriophages. Phylogenetic data do not provide a clear understanding of the origin of these genes in viruses. However, an obvious diversity of viral gene sequences argues against a common ancestor of these genes.

AMG genes are expressed during infection, increasing host energy and resources and redirecting them into virus production [60]. There were some genes in the viromes: *cobS*, *metK*, *DNMT1*, *cysC*, *pbsA1*, etc. The presence of S-adenosylmethionine synthetase (*metK*) suggests that viruses mediate the host response to stress via the production of the Fe–S cluster [61]. In terms of ecology, the ability to modulate synthesis and degradation of Fe–S cluster proteins in viral communities of the photic zone may be important as a means of creating Fe–S clusters that control phage production and reduce host stress, thus maintaining a limited amount of iron in the environment in regions of high primary production. The *cobS* gene (cobaltochelatase) has been identified in all known Myoviridae cyanophages (cyanomyoviruses) [62]. The *cysC* gene (adenylylsulfate kinase), a component of the assimilation pathway for sulphate reduction, is widely used in all three kingdoms of life for the incorporation of sulphide into cysteine [63]. The main tumour suppressor, p53, activated by MDM2 inhibitors, induces the expression of endogenous retroviruses partially through epigenetic factors, histone demethylase (*LSD1*), and DNA-methyltransferase (*DNMT1*) [64]. Triacylglycerol lipase (*TGL*) plays an important role in providing energy for seed germination and plant development. A maize *Zea mays* TGL lipase (ZmTGL) interacts with helper component-proteinase (HC-Pro) of sugarcane mosaic virus (SCMV) during infection, and overexpression of ZmTGL inhibits SCMV infection [65].

In general, the annotation of proteins using three databases has highlighted that the pool of identified proteins is diverse, and that viral replication predominates among the categories, indicating the activity of the viral community. According to the databases, up to 54% (VOG) and 49.8% (PHROG) of the proteins have unknown function, indicating that most viral genes have not yet been characterized. The transcriptomes contained Chaperonin GroEL and gp31 proteins. Chaperonins are protein folding mechanisms found in all cellular forms. Analysis of viral metagenomes showed that the order of large and small subunit genes was linked to the phylogeny of GroEL; both lytic and temperate phages can carry group I chaperonin genes; and viruses carrying the GroEL gene are likely to have large double-stranded DNA (dsDNA) genomes (>70 kb) [66]. The presence of this protein has also been demonstrated when viruses were examined in soil samples [67]. The phage-encoded gp31 protein plays a role in the interaction with the *E. coli* host-encoded GroEL protein and is involved in the proper folding and assembly of the major phage capsid protein, gp23 [68].

Notably, the genomes of RNA viruses and single-stranded DNA are smaller than the genomes of double-stranded DNA viruses [69], which may affect sequence abundance but has no ecological significance. Moreover, the difficulty in obtaining only viral sequences from metatranscriptomes imposes certain problems due to the insufficient depth of the analysed data. For example, in the transcriptomes of the Baltic Sea and Lake Turnetrask (Sweden), the number of identified RNA viruses was much greater in the fraction of 0.8–3 µm than in the fraction of 0.1–0.8 µm, suggesting a larger size of their host [10].

Positive-sense single-stranded RNA viruses, whose genome serves directly as mRNA, can be sequenced together with transcripts that may not reflect their transcriptional activity.

Comparison of metatranscriptomes from various habitats is speculative to some extent due to differences in sample preparation methods. For example, in this study, the extracted RNA was treated with DNase, then reverse transcription and second strand synthesis were performed, followed by sequencing. However, there are methods to increase the yield of RNA sequences belonging to viruses, such as using polyadenylated RNA sequences [70]. Previously, the rRNA reduction approach was shown to provide results consistent with understandings of ecosystem ecology, while the selected poly-A libraries did not provide such results [39]. Recently, a new approach has been developed for sequencing dsRNA viruses that are not extracted in sufficient amounts due to the peculiarities of a standard RNA library preparation, fragmented and primer ligated dsRNA sequencing (FLDS) [71]. The study revealed significant genetic diversity of marine RNA viruses in cellular fractions obtained from surface seawater.

Another difficulty in comparative analysis is the different metadata of the samples: sampling depth, season, and size of the analysed fraction. The results obtained in this study are not consistent with the clustering of DNA viruses performed in our previous study [56]. Thus, the distribution of RNA transcriptomes in clusters did not indicate a dependence on either geographical distance or the trophic status of water bodies, econiches, etc. The only observation that can be made is a cluster with the samples collected during the blooming period (Lakes Seneca, Owasco, Cayuga, Tai, Tiana Beach, and Quantuck Bay), but these data are not sufficient to draw a conclusion. Freshwater lakes such as Seneca (mesotrophic), Cayuga (mesotrophic), and Owasco (more eutrophic than other lakes in the region) [72] are part of the so-called Finger Lakes (11 lakes in total). The RNA libraries were prepared from the 0.2–5 μm size fraction filters. In the study of the samples collected in the estuaries of New York (USA), brown tide blooms were caused by eukaryotic algae, *Aureococcus anophagefferens*, and, in turn, the shift of viral communities was a certain bias of the results during the bloom-free period, so the results depend on the sampling period. Samples were collected on 0.2 µm polycarbonate filters, and RNA was pre-treated by rRNA reduction [39]. *Microcystis aeruginosa* also predominated in the study of Lake Tai during summer sampling in 2014. Phages, for which Microcystis was a host, accounted for 47.9% of the total number of viral reads. In this study, the viral fraction was used, i.e., lake water was passed through the 0.2 µm filter [38]. A sample from the Yangshan deep-water harbour, located in the freshwater–seawater mixing zone, was the most distant branch [12]. Most likely, this distribution was due to various methods, and for a comparative analysis, samples analysed with the same method and similar treatment algorithm should be used, otherwise the true picture cannot be elucidated.

It is likely that comparison by transcripts does not lead to clustering by trophicity, salinity, and belonging to marine or freshwater ecosystems, which may be due to many factors affecting replication at a given time.

In terms of ecology, it is important to take into account the dynamics of viral activity, which was done when the water in the North Pacific Ocean was studied. Water samples were collected and sampled every four hours for ~2.6 days. Viruses infecting the most common taxa often had shared transcriptional activity synchronised with putative hosts [40]. For several months, the dynamics of viral transcripts were examined in Finger Lakes [11] and Lake Tai [38]. The subsequent study of metatranscriptomes in Lake Baikal certainly requires such an approach.

One of the most important applications of DNA and RNA sequencing for the studies of Lake Baikal is associated with assessing the ecosystem health of this world’s largest lake. The data obtained from sequencing can be used to quickly and relatively inexpensively detect viruses that threaten human health. This study may serve as a reference for determining future fluctuations in taxonomic composition and transcriptional activity of viruses during summer.

## 5. Conclusions

In this study, we analysed for the first time the viral fraction of metatranscriptomes from Lake Baikal. The identified transcripts confirmed the previous assumption of high replication activity of bacteriophages in the lake based on DNA viromes. We detected sequences similar to those of RNA viruses and determined five families according to the RefSeq database. UPGMA analysis showed that transcriptomes from Lake Baikal formed a separate cluster and differed from known transcriptomes. Comparative analysis with other ecosystems did not reveal any pattern, which we believe is related to different methods of RNA extraction and transcriptome sequencing. The transcripts contained genes similar to those of the giant viruses *Pithovirus sibericum*, *Cedratvirus A11*, and Yellowstone Lake phycodnavirus, indicating the presence of related genotypes in Lake Baikal.

## Figures and Tables

**Table 1:** Identified proteins similar to *Pithovirus sibericum* and *Cedratvirus A11* (Nucleocytoviricota) according to the RefSeq database.

*Pithovirus sibericum*		
Protein	Accession	e-value
Glycosyltransferase	YP_009000961	3.1 × 10^−21^
Adenylosuccinate synthetase	YP_009000992	4.2 × 10^−79^
DNA-binding ferritin-like protein	YP_009001267	1.8 × 10^−17^
ABC2 type transporter superfamily protein	YP_009001225	1.2 × 10^−17^
PDZ serine protease	YP_009001035	2.9 × 10^−9^
pv_324	YP_009001226	3.0 × 10^−12^
Ser/Thr protein kinase	YP_009001306	1.2 × 10^−12^
Glycosyltransferase family 2	YP_009001307	9.9 × 10^−10^
Deoxycytidine triphosphate deaminase	YP_009001173	4.1 × 10^−10^
Ribonucleoside-diphosphate reductase	YP_009001342	1.4 × 10^−8^
Formamidopyrimidine-DNA glycosylase	YP_009001363	2.3 × 10^−15^
Ran-like GTP-binding protein	YP_009001029	7.6 × 10^−12^
***Cedratvirus A11***		
**Protein**	**Accession**	**e-value**
D-3-phosphoglycerate dehydrogenase, type2	YP_009328950	1.7 × 10^−99^
DNA-directed RNA polymerase subunit RPB2	YP_009329295	2.4 × 10^−14^
Adenylosuccinate synthetase	YP_009329210	7.8 × 10^−14^
dTDPD-glucose 4,6-dehydratase	YP_009329097	4.2 × 10^−11^
ABC2 type transporter superfamily protein	YP_009329403	3.2 × 10^−26^
NAD dependent epimerase/dehydratase	YP_009329336	9.8 × 10^−19^
Translation elongation factor EF-1 subunit alpha	YP_009329269	3.2 × 10^−26^
Macrocin O-methyltransferase	YP_009329463	6.4 × 10^−25^
PD-(D/E)XK nuclease	YP_009329328	2.5 × 10^−17^
Hexapeptide transferase	YP_009329047	4.9 × 10^−12^
5′nucleotidase/apyrase	YP_009329013	8.0 × 10^−18^
Putative serine/threonine-protein kinase/receptor	YP_009329205	9.7 × 10^−10^

**Table 2:** AMG genes identified in metatranscriptomes of Lake Baikal.

AMG KO Name	AMG KO	RVP1	RVP2	RVP3	Enzyme	Pathway
*GCH1*	K01495	1	0	0	GTP-cyclohydrolase	Folate biosynthesis
*gmhC*	K03272	2	0	0	D-beta-D-heptose 7-phosphate kinase	Lipopolysaccharide biosynthesis
*queE*	K10026	1	1	0	7-carboxy-7-deazaguanine synthase	Folate biosynthesis
*pbsA1*	K21480	1	0	0	heme oxygenase	Porphyrin metabolism
*GAOA*	K04618	1	0	0	galactose oxidase	Galactose metabolism
*glf*	K01854	1	0	0	UDP-galactopyranose mutase	Galactose metabolism
*SCD*	K00507	1	0	0	stearoyl-CoA desaturase	Biosynthesis of unsaturated fatty acids
*kdsD*	K06041	1	0	0	arabinose-5-phosphate isomerase	Lipopolysaccharide biosynthesis
*lpxH*	K03269	1	0	0	UDP-2,3-diacylglucosamine hydrolase	Lipopolysaccharide biosynthesis
*NAMPT*	K03462	1	0	0	nicotinamide phosphoribosyltransferase	Nicotinate and nicotinamide metabolism
*cysC*	K00860	1	0	0	adenylylsulfate kinase	Purine metabolism
*P4HA*	K00472	1	0	0	prolyl 4-hydroxylase	Arginine and proline metabolism
*TGL2*	K01046	0	1	0	triacylglycerol lipase	Glycerolipid metabolism
*gpmB*	K15634	0	1	0	2,3-bisphosphoglycerate-dependent phosphoglycerate mutase	Glycine, serine, and threonine metabolism
*DNMT1*	K00558	0	1	0	DNA (cytosine-5)-methyltransferase 1	Cysteine and methionine metabolism
*cobS*	K09882	0	0	1	cobaltochelatase CobS	Porphyrin metabolism
*metK*	K00789	0	0	1	S-adenosylmethionine synthetase	Cysteine and methionine metabolism

## Data Availability

Raw fastq files were deposited into the Sequence Read Archive (SRA) NCBI under the project number PRJNA824673.

## Supplementary Materials

The following supporting information can be downloaded at: https://www.mdpi.com/article/10.3390/microorganisms10101937/s1, Figure S1: Ratio of functional categories for the RVP1, RVP2, and RVP3 viromes according to the PHROG database; Figure S2: Heat map created using orthologous proteins from the PHROG database, which were similar to the proteins from this study; Table S1: Functional annotation of viral proteins using VOGDB.
