# Supplementary material for: The Viral Fraction Metatranscriptomes of Lake Baikal
Source: Microorganisms. 2022 Sep 29;10(10):1937. doi: 10.3390/microorganisms10101937 (PMC9611531; doi:10.3390/microorganisms10101937)
Supplement: Supplementary file 1 [file microorganisms-10-01937-s001.zip › microorganisms-1847373 - Supplementary Figures.pdf]

microorganisms

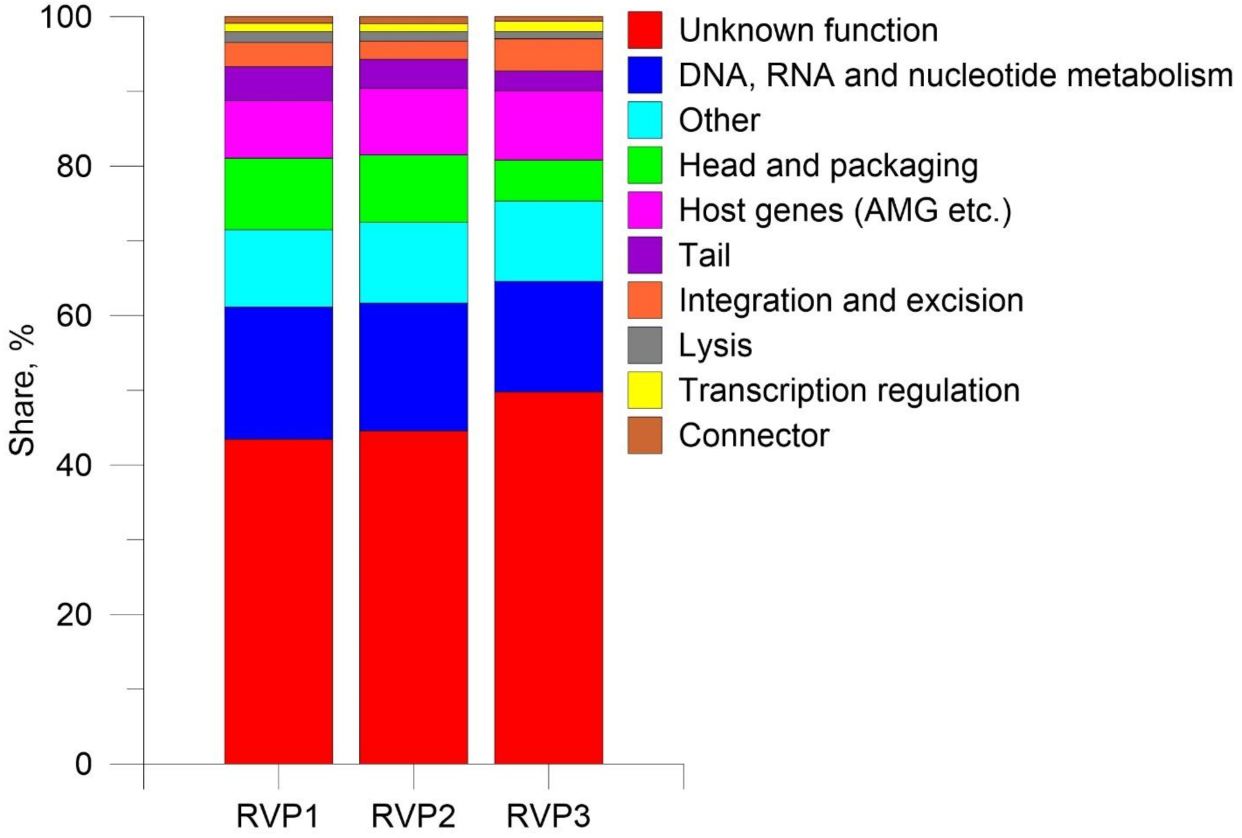

Figure S1. Ratio of functional categories for the RVP1, RVP2 and RVP3 viromes according to the PHROG database.

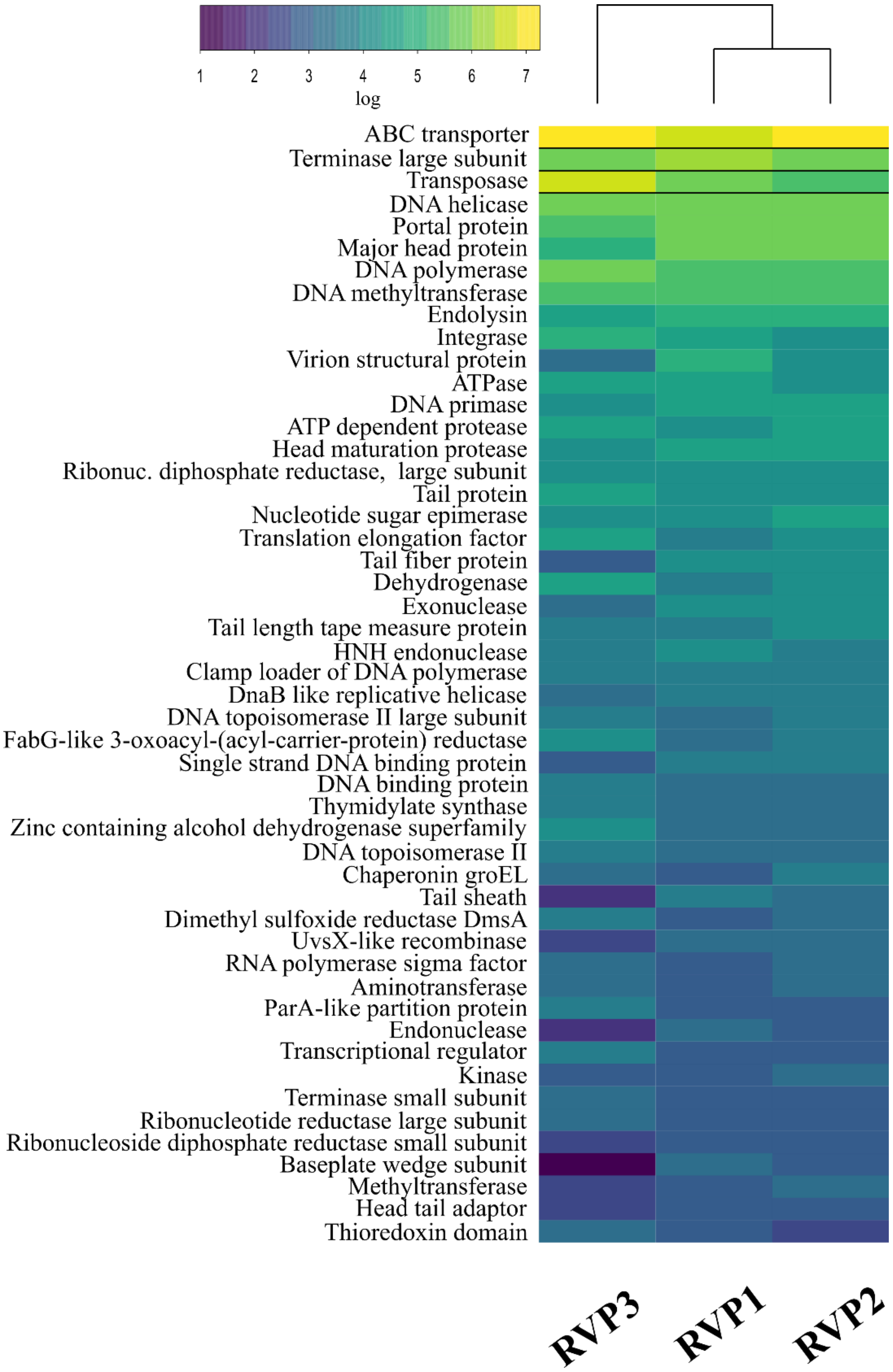

Figure S2. Heat map created using orthologous proteins from the PHROG database, which were similar to the proteins from this study.
